# First Member of ‘Higher Endomychidae’ (Coleoptera: Coccinelloidea) from the Mid-Cretaceous Amber of Myanmar and New Insights into the Time of Origin of the Handsome Fungus Beetles †

**DOI:** 10.3390/insects13080690

**Published:** 2022-07-31

**Authors:** Wioletta Tomaszewska, Karol Szawaryn, Emmanuel Arriaga-Varela

**Affiliations:** Museum and Institute of Zoology, Polish Academy of Sciences, Wilcza 64, 00-679 Warsaw, Poland; k.szawaryn@gmail.com (K.S.); arriagavarelae@gmail.com (E.A.-V.)

**Keywords:** burmite, Cenomanian, fossil, handsome fungus beetles, new genus, new species, endomychine complex

## Abstract

**Simple Summary:**

We discovered an extinct handsome fungus beetle almost 100 million years old embedded in amber from Myanmar. Comparing and analyzing the characteristics of its body with those of other beetles of this family living today, we were able to find out which ones could be their closest relatives. What we discovered suggests that our beetle, representing a new genus and species, is a part of a group called “Higher Endomychidae”. This group shares some features with members of the family Coccinellidae, the well-known ladybugs or ladybirds. Our finding supports the hypothesis that handsome fungus beetles (family Endomychidae) originated at least at the beginning of the Cretaceous period, and most probably in the Jurassic, coinciding with the heyday of dinosaurs on earth.

**Abstract:**

A new genus and species of the family Endomychidae (Coleoptera: Coccinelloidea): *Cretostenotarsus striatus* Tomaszewska, Szawaryn and Arriaga-Varela **gen. et sp. nov.** are described, diagnosed and illustrated from the mid-Cretaceous amber from northern Myanmar. To test the systematic placement of the new extinct genus and species within the family, a phylogenetic analysis was conducted. A dataset of 38 morphological characters scored for 29 species (including the new fossil taxon), members of Endomychidae *sensu stricto* and representatives of Coccinelloidea as outgroups were analyzed using maximum parsimony. The results of the analysis indicate unequivocally that *Cretostenotarsus striatus* is a member of the *Stenotarsus* clade within a monophyletic ‘endomychine complex’ *sensu* Robertson et al. (2015), which corresponds to ‘Higher Endomychidae’ *sensu* Tomaszewska (2005). The present discovery confirms at least the Jurassic origin of Coccinelloidea and indicates a much older origin of Endomychidae than previously hypothesized.

## 1. Introduction

The family Endomychidae (handsome fungus beetles) is a moderately diverse family of mycophagous beetles that has been a subject of a major taxonomic rearrangement in the last decade. Traditionally, Endomychidae contained 12 subfamilies [1] and was classified in the superfamily Cucujoidea [2,3,4,5], in the derived group called ‘Cerylonid Series’ [6]. However, the most recent, comprehensive molecular research on Cucujoidea by Robertson et al. [7] has resulted in the formal recognition of the Cerylonid Series as an independent superfamily Coccinelloidea and the redefinition of Endomychidae by removing Anamorphidae, Mycetaeidae, and Eupsilobiidae as separate families.

Endomychidae currently contains over 1600 described species classified in about 90 genera distributed in all zoogeographical realms, with the highest diversity in the tropical and subtropical regions of the world [1,3,4]. The study of Robertson et al. [7] recovered two main clades within the family, the ‘merophysiine complex’ and the ‘endomychine complex’. The merophysiine complex includes the subfamilies Leiestinae, Merophysiinae, and Pleganophorinae, the basal lineages of the family according to Tomaszewska [4], while the endomychine complex includes Cyclotominae, Endomychinae, Epipocinae, and Lycoperdininae, and corresponds to ‘Higher Endomychidae’ *sensu* Tomaszewska [4]. The endomychine complex is supported morphologically by the pseudotrimerous tarsi in adults and the V- or U-shaped frontal arms on the head, and four pairs of stemmata in larvae [4]. The study of Robertson et al. [7], however, did not include exemplars of subfamilies Danascelinae and Xenomycetinae, this last one a sister group to ‘Higher Endomychidae’ in the analysis of Tomaszewska [4], so their relationships with the rest of the handsome fungus beetles remain unclear. Moreover, some current subfamilies, e.g., Endomychinae in the new sense, which now includes the genus *Endomychus* plus all genera of the former subfamily Stenotarsinae, are an anatomically heterogeneous group with no potential supporting synapomorphies. All this makes the phylogeny and evolution of the group still unclear and the classification of the family preliminary, requiring further molecular and morphological research, including fossil data.

Fossil taxa of handsome fungus beetles have been poorly known to date. Only 14 species of Endomychidae *sensu stricto* have been formally described to the present. Among them, eight species, representatives of subfamilies Leiestinae, Merophysiinae, Pleganophorinae, and Endomychinae, have been described from Cenozoic ambers (Eocene Baltic, Bitterfeld, and French amber from Oise) [8,9,10]. Even more intriguing and systematically significant fossil Endomychidae, important for elucidating the origin and early evolution of the family, come from Mesozoic deposits. Recently, Tomaszewska et al. [11] described the new genera *Cretolestes*, *Burmalestes* (Leiestinae), *Cretoparamecus* (Merophysiinae), and *Palaeomycetes* (Xenomycetinae), and Li et al. [12] described the genus *Rhomeocalpsua* (of *incertae sedis* placement in Endomychidae), the oldest known endomychids from northern Myanmar amber, known as the Burmese amber or burmite which contains an outstandingly diverse biota from the early Upper Cretaceous (approx. 99 Ma) [13].

Noticeable is the diversity of extinct forms in the subfamilies belonging to the basal lineages of Endomychidae [4]. Apart from described taxa, additional undescribed representatives of Leiestinae and Merophysiinae are abundant in the Myanmar amber, and represent new species and new genera (WT and EAV, personal observation). This can suggest at least an Early Cretaceous origin of Endomychidae ancestor, which agrees with McKenna et al. [14] molecular study and estimates indicating that Coccinelloidea appeared during the Middle Jurassic.

However, recent observations on additional specimens embedded in Myanmar amber have suggested the existence of a member of the more derived evolutionary lineage ‘Higher Endomychidae’, with overall similarity to *Stenotarsus* and its allies, at that time. This discovery would suggest older than previously hypothesized, presumably Jurassic origin of Endomychidae, agreeing with results of, e.g., Cai et al. [15], indicating that Coccinelloidea diverged in the Late Triassic to Early Jurassic.

Most endomychid subfamilies, as defined by the molecular analyses of Robertson et al. [7], are also supported by synapomorphies recovered in the studies of Tomaszewska [3,4]. Nevertheless, some groups, like Endomychinae, are difficult to characterize morphologically. Moreover, the synapomorphies for some subfamilies, e.g., those belonging to the endomychine complex (‘Higher Endomychidae’), like Epipocinae, Cyclotominae, and former Stenotarsinae are difficult or impossible to observe in fossil specimens, as they refer to the structures of male and female genitalia. Therefore, to avoid ambiguity and a narrative/subjective assignment of the new fossil taxon within the family, cladistic analysis of morphological data was conducted, with non-visible morphological characters of the fossil taxon scored as ‘?’.

In the present contribution, we describe and illustrate a new genus and species from the Upper Cretaceous Myanmar amber. Based on the results of our analyses, the new taxon is classified in the subfamily Endomychinae as currently defined [7]. Our discovery represents the oldest fossil taxon of the endomychine complex *sensu* Robertson et al. [7] (=’ Higher Endomychidae’ *sensu* Tomaszewska [4]), thus filling an important gap in our knowledge of the early evolution of the Endomychidae. Completing the gaps in our knowledge of the fossil taxa of handsome fungus beetles will contribute to future, objective fossil-based calibration in phylogenetic analyses (EAV et al. in preparation) and gradually bring us closer to getting a complete picture of the origin and evolution of the Endomychidae and the Coccinelloidea superfamily.

## 2. Materials and Methods

### 2.1. Examination and Deposition of Fossil Taxon

The study is based on a specimen of Endomychidae embedded in the Myanmar amber originated from the Kachin State of northern Myanmar. The age of these amber deposits is generally considered the earliest Cenomanian [16] or possibly the latest Albian [17], thus, near the boundary between Upper and Lower Cretaceous. U-Pb zircon dating conducted by Shi et al. [13] restricted its age at 98.79 ± 0.62, which is equivalent to the earliest Cenomanian, Upper Cretaceous. The type specimen of the new species described here is deposited in the Museum of Amber Inclusions at the University of Gdańsk, Poland (MAIG), and is in accordance with all ethical standards for studying fossils from Myanmar [18].

The amber was cut, ground, and polished prior to the study. The specimen was examined and documented with a Leica M205A stereomicroscope with a Leica DM6000 digital camera operating under Leica Application SuiteVR LAS 3.7 (MAIG). The following measurements were made and are used in the description: total length—from the apical margin of clypeus to apex of elytra; pronotal length—from the middle of anterior margin to the margin of basal foramen; pronotal width—at widest part; elytral length—across sutural line including scutellum; elytral width—across both elytra at the widest part. The morphological terminology and the generic attribution and comparison with extant and extinct taxa follow Tomaszewska [3,4,5]. 

Taxonomic acts established in the present work have been registered in ZooBank (see below), together with the electronic publication LSID: urn:lsid:zoobank.org:pub: 37F51B30-D88E-461D-852B-DBBAECF1024D.

### 2.2. Morphological Dataset and Phylogenetic Analysis

The main aim of the cladistics analysis of morphological characters conducted during this study was a recognition of a correct subfamily/genus group placement of the new fossil genus and species within the family Endomychidae. Despite some contradictions between the results of analyses based solely on molecular data [7] or morphology [3,4], having at disposal a somewhat limited set of morphological characters of a new taxon from the past, the cladistics analysis of morphological characters was the most objective way to recognize closest relatives of the newly described extinct genus and species.

Taxon sampling and characters used for the analysis are based in a broad sense on Tomaszewska [3,4], with some modifications made in an attempt to provide resolution among genera of Endomychidae *sensu stricto*. Representatives of Endomychidae, Eupsilobiidae, Mycetaeidae, and Coccinellidae were sampled, including the type species for genera when specimens were available for study. A member of Hobartiidae, *Hobartius eucalypti* (Blackburn), was used as a more distant outgroup. In total, our morphological data matrix comprised 29 taxa (22 ingroup taxa including new fossil and seven outgroups), scored for 38 multistate characters. Unknown character states were treated as missing and coded with ‘?’. The list of morphological characters and the full character matrix can be found in Appendix A and Appendix B, respectively.

The maximum parsimony (MP) analysis of our dataset was conducted in TNT 1.5 [19] to find the most parsimonious trees (MPTs), using the New Technology (NT) option, using Driven Search with Sectorial Search, Ratchet, and Tree fusing options activated with standard settings; and the Traditional Search (TS) option under the following parameters: memory set to hold 1,000,000 trees, tree bisection—reconnection (TBR) branch-swapping algorithm with 1000 replications saving 100 trees per replicate; zero-length branches collapsed after the search. All characters were treated as unordered, and analysis was performed under equal weights. Bremer support was calculated using the TNT Bremer function, using suboptimal trees up to 20 steps longer. Character mapping was done in Winclada v1.00.08 [20] using unambiguous optimization.

## 3. Results

### 3.1. Phylogenetic Analysis of Morphological Dataset 

**Figure 1:**
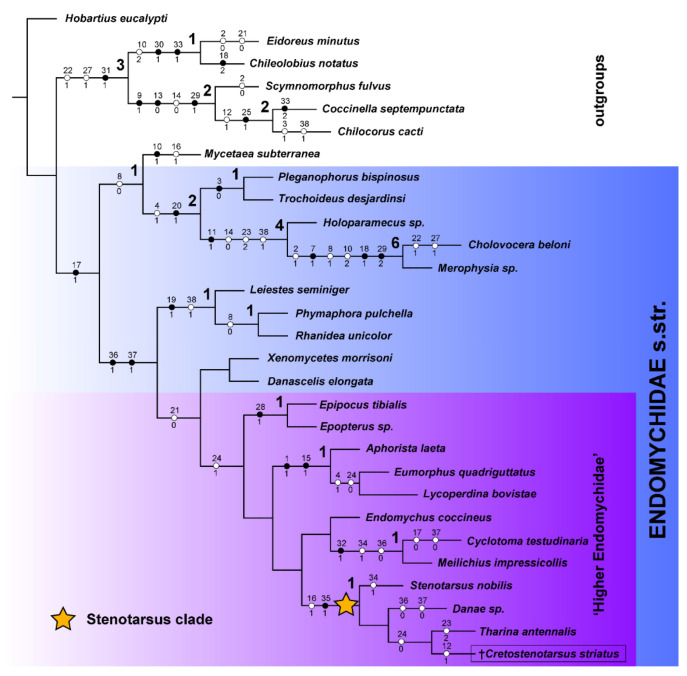
Single maximum parsimony tree based on morphological matrix obtained in TNT. Bremer support values are shown over the nodes. Fossil *Cretostenotarsus* species indicated with ‘†’.

The phylogenetic analysis conducted under different search strategies resulted in sets of at least partly similar resolution. The maximum parsimony analysis under New Technology Search (MP NT) resulted in a single most parsimonious tree (MPT) with a length (L) of 87 steps, consistency index (CI) = 52, and retention index (RI) = 75 (Figure 1). 

The MP analysis under Traditional Search (MP TS) resulted in 16 MPTs with some trees displaying the same or similar resolution as the single tree from the NT option. However, a strict consensus tree calculated from the 16 MPTs from the TS (L = 97 steps; CI = 47; RI = 68) displays almost all recognized subfamilies used in the analysis, mostly as unresolved polytomy (Appendix C). The sister relationships of Eupsilobiidae + Coccinellidae and *Mycetaea* + (Pleganophorinae + Merophysiinae), as well as fossil taxon recovered as part of the *Stenotarsus* clade, are recovered in all trees from TS and the tree from NT analysis.

From the obtained resolutions, we selected as our preferred tree the best-resolved topology—the single tree from the MP NT search (Figure 1), which mostly agrees with Tomaszewska [4] and at least partly agrees with Robertson et al. [7] results.

Our results of morphology-based analysis are in contradiction with the current, molecular-based classification of Endomychidae [7] in a few aspects: (1) The family Endomychidae+ is recovered as a monophyletic group, including Mycetaeidae while Mycetaeidae is recovered as sister group to Eupsilobiidae + Coccinellidae in Robertson et al. [7]; (2) Leiestinae recovered as sister group to a clade comprising Xenomycetinae, Danascelinae and “Higher Endomychidae”, while Leiestinae is a sister group to Pleganophorinae + Merophysiinae in Robertson et al. [7]; and (3) *Endomychus coccineus* recovered as sister group to Cyclotominae, and not forming a clade with former Stenotarsinae, what makes the currently recognized subfamily Endomychinae *sensu* Robertson et al. [7] polyphyletic.

The group of ‘Higher Endomychidae’ *sensu* Tomaszewska [4] (=endomychine complex *sensu* Robertson et al. [7] is recovered in our analysis, as shown in Figure 1.

The present results seem to be morphologically well justified. However, we are aware of the limitation of morphology-only analysis. Therefore we do not intend to change current classifications, leaving the above problems, particularly the monophyly of Endomychinae, to further research with an integrative approach. For the present paper, we follow the current classification and name the clade containing the former Stenotarsinae genera as subfamily Endomychinae. 

The following taxonomical decision is based on the preferred tree.

### 3.2. Systematic Palaeontology

Order Coleoptera Linnaeus, 1758

Suborder Polyphaga Emery, 1886

Superfamily Coccinelloidea Latreille, 1807

Family Endomychidae Leach, 1815

Subfamily Endomychinae Leach, 1815

***Cretostenotarsus* gen. nov.**

*LSID*. urn:lsid:zoobank.org:act: 6DEC503D-BF61-48F9-9202-BA6A5B92FBFF.

**Derivation of name.** The name of the new genus is a combination of the Creto-, referring to the Cretaceous age of the deposit with the name *Stenotarsus*, the genus group where the new taxon belongs. The gender is masculine.

**Composition.** The new genus is monotypic, represented by the type species only.

**Type species.** Cretostenotarsus striatus sp. nov.

**Figure 2:**
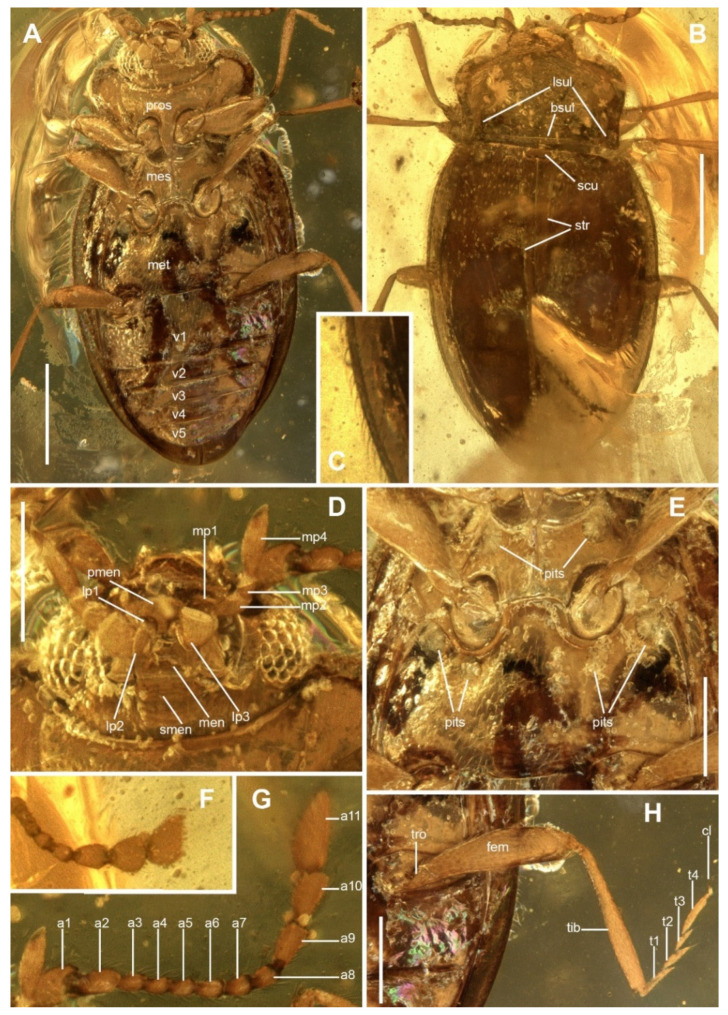
*Cretostenotarsus striatus* sp. nov., holotype, MAIG 5999. (**A**), habitus, ventral. (**B**), habitus, dorsal. (**C**), detail of lateral margin of elytra with setae. (**D**), head and mouthparts, ventral. (**E**), meso- and metaventrite, ventral. F, antennal club, dorsal. (**G**), antenna, ventral. (**H**), hind leg. Abbreviations: a1–a10, antennomeres; bsul, basal sulcus; cl, tarsal claw; fem, femur; lp1–lp3, labial palpomeres; lsul, lateral sulci; men, mentum; mes, mesoventrite; met, metaventrite; mo1–mp4, maxillary palpomeres; pment, prementum; pros, prosternum; smen, submentum; str, sutural striae; t1–t4, tarsomeres; tib, tibia; tro, trochanter; v1–v5, ventrites. Scale bars represent: 500 µm (**A**); 250 µm (**D**,**E**,**H**).

**Diagnosis.***Cretostenotarsus* can be distinguished from all known extant Endomychinae genera by the following combination of characters: body comparatively small, oval, pubescent (Figure 2C), with irregularly punctate elytra; pronotum weakly narrower than the base of elytra (Figure 2B), widely margined laterally, with anterior angles rounded not produced, with distinct basal and triangular lateral sulci; sutural striae complete throughout the elytra (Figure 2B); scutellar shield transverse, semi-rectangular; and tarsi 4-4-4, simple with tarsomeres 1 and 2 weakly lobed (Figure 2H).

The new genus most resembles the extant genera *Stenotarsus* Perty, 1832, and *Chondria* Gorham, 1887, in overall body shape (oval with elytra rounded laterally and pronotum weakly narrower than the base of elytra). However, elytra with sutural striae, scutellar shield short and transverse, anterior angles of pronotum rounded and not produced, and terminal labial palpomere at most as long as wide and widening apically separate *Cretostenotarsus* from both genera. Additionally, simple tarsomeres distinguish it from *Stenotarsus*.

Moreover, *Cretostenotarsus* can be distinguished from the genus *Danae* Reiche, 1847 by having a more oval body, the pronotum without produced anterior angles and with deeply impressed sulci, and by five abdominal ventrites; from *Saula* Gerstaecker, 1858 and *Africanasaula* Pic, 1946 by a more oval body, pronotum weakly narrower than base of elytra, widely margined laterally with deeply impressed lateral and basal sulci, and five abdominal ventrites; from *Tragoscelis* Strohecker, 1953 by having not as stout antenna and not produced and acute front angles of pronotum, and by having lateral margins of elytra flattened and well visible from above; from *Ectomychus* Gorham, 1887 by a more oval body, symmetrical antennal club, distinct/deep basal sulcus on the pronotum and not produced anterior pronotal angles; from *Perrisina* Strand, 1921 by light brown body, terminal labial palpomere short and widening apically, and pronotal lateral margins not so strongly elevated; from *Paniegena* Heller, 1916 by uniformly light brown body and elytra irregularly and finely punctate; from *Tharina* Arriaga-Varela et al., 2018 by the differently shaped mouthparts (maxillary and labial structures), well developed pronotal sulci, prosternal process narrowing towards acute apex, and posterior angles of pronotum regular without indentation; and from *Endomychus* Panzer, 1795 by the distinctly pubescent body, different shape of scutellar shield, pronotum widely margined laterally, sutural striae on the elytra, much more elongate terminal maxillary palpomere, ligula widening laterally and not elongate terminal labial palpomere.

*Cretostenotarsus* is distinguished from the Cenozoic fossil member of the subfamily Endomychinae from Baltic amber, *Zemyna* Tomaszewska, 2018 (=*Laima* Alekseev & Tomaszewska, 2018) by having a brown, oval body, pronotum only weakly narrower than the base of elytra with distinct triangular lateral sulci, and sutural striae complete throughout the elytra.

***Cretostenotarsus striatus* sp. nov.** (Figure 2A–H)

*LSID*. urn:lsid:zoobank.org:act:99D602C3-01AF-4C63-B355-84A7D9C650C5 

**Derivation of the name.** The species name refers to sutural striae on the elytra, present very rarely in the subfamily.

**Holotype.** MAIG No. 5999, sex unknown. The holotype is embedded in a small, flattened, subquadrate piece (8.0 × 8.3 × 3.0 mm) of clear-yellow colored Myanmar amber (burmite). Complete specimen. Syninclusions are present in the form of specimens of Diptera and Hymenoptera and several small organic pieces.

**Diagnosis**. As stated for the new genus.

**Description.** Body length 2.25 mm; width (at the widest point in about the middle part of elytra) 1.14 mm. Body oval (about 1.97 times as long as wide) and moderately convex, brown (Figure 2B); dorsal surfaces shiny and distinctly covered with moderately long, pale pubescence (Figure 2C).

Head transverse, retracted into prothorax to the hind margin of eyes, finely punctate and pubescent. Clypeus transverse and flat. Eyes large, rounded, prominent, and coarsely faceted (Figure 2D). Antennal insertions visible from above. Antenna 11-segmented (Figure 2G), relatively long, extending well behind bases of elytra, about as long as the width of pronotum; scape stout, distinctly larger than pedicel; antennomeres 3–8 subequal, regular, scarcely widening from their bases towards apices, each about 1.6 times as long as wide, about 0.5 times as long pedicel; antennomeres 9–11 form elongate, narrow, loose scarcely flattened club (Figure 2F); antennomeres 9 and 10 about the same length, and antennomere 11 slightly longer, obliquely truncate at apex. Maxillary palpomeres with membranous insertions on their inner apical edges (Figure 2D); palpomere 1 shortest, palpomere 2 longer than 1 and 3, about 2 times longer than 3; terminal palpomere longer than remaining palpomeres combined, narrow at the base, widest toward middle length then tapering and weakly obliquely truncate apically, about 2.8 times as long as wide in the widest part. Labium with mentum transverse, widest near the base; prementum transverse with ligula distinctly widened laterally; labial palpomeres moderately widely separated; palpomere 1 shortest/smallest; palpomere 2 transverse; terminal palpomere scarcely widened towards the apex, about as long as wide, nearly 2 times longer than palpomere 2, truncate at apex.

Prothorax 0.57 times as long as wide (Figure 2B); widest near anterior third, slightly constricted at basal third; disc rather finely punctate with interspaces about 1.5–2.5 diameters apart. Lateral sides widely margined; anterior angles rounded and not produced, posterior angles nearly right-angled; disc evenly convex; lateral sulci in the form of triangular, concave impressions, basal sulcus well developed. Posterior margin nearly straight, anterior margin scarcely rounded medially. Prosternal process moderately widely separating procoxae (Figure 2A), extending posteriorly distinctly beyond them, narrowing continuously towards the apex, which is almost acute. Procoxae circular in outline (Figure 2A). Prosternum with anterior margin straight; in front of coxae about 0.8 times as long as longitudinal coxal diameter.

Mesoventrite with two large oval pits on sides near anterior margin (Figure 2E); with longitudinal ridge medially and weak concavities on its sides; mesocoxal cavities circular, widely separated with mesoventral intercoxal process about 1.35 times as wide as coxal diameter; mesocoxal cavities open laterally; exposure of mesotrochantin is uncertain. Scutellar shield moderately large (Figure 2B), strongly transverse, about 2.9 times wider than long, and of semi-rectangular shape. Elytra 1.42 times as long as wide, 1.43 times as wide as pronotum; moderately convex; lateral margins moderately widely flattened, visible from above; irregularly punctate, punctures sparser than those on head and pronotum, about 2.0–4.0 diameters apart; sutural stria distinct, entire; epipleuron narrow (Figure 2A,H), incomplete at the apex. Metaventrite transverse, nearly 1.7 times as broad as long (Figure 2A,E); more than twice longer than mesoventrite; distinctly bordered anteriorly; intercoxal process straight between mesocoxae; postcoxal pits two pairs, large. Metacoxae transverse, broadly separated (Figure 2A,H). Hind wings not visible.

Legs comparatively long (Figure 2A,H). Femur subclavate, pubescent. Tibiae weakly widening towards apices without modifications, densely covered with short pubescence; tibial spurs absent. Tarsal formula 4-4-4 (Figure 2H). Tarsi long, tarsomeres 1–3 gradually slightly shorter and weakly lobed; tarsomere 4 slightly longer than tarsomeres 2–3 combined.

Abdomen with five ventrites (Figure 2A); ventrite 1 weakly shorter than the remaining four ventrites combined. Ventrites 2, 3, and 4 subequal in length; terminal ventrite rounded apically. Postcoxal lines absent. Abdominal punctation similar to those on metathorax.

**Locality and horizon.** Myanmar, Kachin State, Hukawng Valley, Myanmar amber; unnamed horizon; Cretaceous, Upper Albian to Lower Cenomanian.

## 4. Discussion

Knowledge of fossil faunas is crucial for making conclusions about the origin and evolution of studied groups. Although the fossil fauna of handsome fungus beetles is still poorly known, the situation has improved constantly, especially in recent years.

Representatives of Endomychidae *sensu lato* in the fossil record have appeared in several publications [1,21,22,23,24,25,26,27], but only very few Cenozoic taxa have been properly named and formally described until recently [8,9,10]. Among 16 genera of Endomychidae *sensu lato* from the Eocene reported in the literature, only 13 species are formally described, 12 from the Baltic and Bitterfeld ambers (ca. 40–45 Ma), and one species from the French amber from Oise (ca. 53 Ma). Many of them are recently described members of Anamorphidae from the Eocene Baltic and Bitterfeld amber, one classified in *Symbiotes* Redtenbacher, and four in extinct monotypic genera: *Gramboale*, *Kleinzaches*, *Palaecoryphus,* and *Giltine* [9]. Some species of Endomychidae from the Eocene Baltic amber have been, at least initially, assigned to extant genera, e.g., *Trochoideus*, *Holoparamecus*, *Mycetina,* or *Lycoperdina*. Mycetaeidae are only known through an unnamed *Mycetaea* species reported by Klebs [24], and Eupsilobiidae lack any reported fossil species.

Of the properly described taxa of Endomychidae *sensu stricto* from the Eocene, *Palaeoestes* Kirejtshuk & Nel (French Oise amber), *Phymaphoroides* Motschulsky, and *Glesirhanis* Shockley & Alekseev (Baltic amber) represent extinct genera of Leiestinae and *Zemyna* Tomaszewska (=*Laima* Alekseev & Tomaszewska) (Baltic amber) is the only known extinct genus of the subfamily Endomychinae. Two species of *Trochoideus* described by Alekseev and Tomaszewska [9] are the only Eocene species classified in the extant genus of the subfamily Pleganophorinae. While two species of *Holoparamecus* described by Reike et al. [10] represent the only Eocene species of this extant genus of the endomychid subfamily Merophysiinae (although treated as family Merophysiidae by these authors, ignoring the results of numerous research based on morphological and molecular data sets (e.g., [4,5,7])).

Nearly all those discoveries that appeared over 170 years since the first record of fossil Endomychidae [21] originally seemed to confirm Carpenter’s suggestion that the family Endomychidae *sensu lato* originated during the Eocene [27]. Only the description of *Palaeoestes eocenicus* Kirejtshuk & Nel, a member of the subfamily Leiestinae from French amber (53 Ma) [28], changed this situation, suggesting an older origin of the group, probably in the Paleocene (65–54.8 Ma). First estimates of the Mesozoic origin of the Endomychidae by Ponomarenko [29] indicated that the group originated during the Late Cretaceous. And subsequent reports of Endomychidae in Myanmar (99 Ma) and Lebanese amber (125 Ma) by Poinar and Poinar [30] further supported a much older origin for the family.

The recent discovery and the formal description of the Endomychidae *sensu stricto* members in Myanmar amber confirmed at least the Cretaceous origin of Endomychidae [11]. Tomaszewska et al. [11] described the oldest known endomychids from Myanmar amber, representatives of the subfamilies Leiestinae (*Cretolestes*, *Burmalestes*), Merophysiinae (*Cretoparamecus*), and Xenomycetinae (*Palaeomycetes*), all belonging to the basal evolutionary lineages of the handsome fungus beetles [4].

The species (described and undescribed) known to date from the Cretaceous Myanmar amber display much greater taxonomic diversity than the taxa from the Eocene ambers described so far. Particularly noticeable is the diversity of extinct forms in the subfamily Leiestinae in the Myanmar amber. The diversity and distribution of this group seem to have decreased in time, with a few members known from Eocene amber and currently being a small and exclusively Holarctic group [3,31]. While the subfamily Xenomycetinae is not recorded from the Eocene deposits until now. The occurrence of Merophysiinae in the Myanmar amber may be correlated with the increased availability of termite and ant colonies inhabited by many species of this subfamily. However, the species of *Trochoideus*, a member of Pleganophorinae, likely a sister group to Merophysiinae, being the typical symphile, is known only from the Eocene so far.

The abundance and diversification of the basal Endomychidae in the mid-Cretaceous environments suggest at least the Early Cretaceous origin of this group, which agrees with McKenna et al. [14] molecular study indicating the Middle Jurassic (187–157 Ma) origin of the superfamily Coccinelloidea.

However, some other, more recent estimates suggest that Coccinelloidea diverged in the Late Triassic to Early Jurassic (217–182 Ma), with most extant families originating in the Jurassic to Early Cretaceous [15], or even this superfamily could occur in mid-Permian to mid-Triassic (ca. 267–238 Ma) [32]. And our current discovery of a member of a more derived evolutionary lineage of Endomychidae in Upper Cretaceous amber seems to confirm these estimates and indicate the Jurassic origin of the family.

## 5. Conclusions

The good preservation and visibility of the fossil specimen from Myanmar amber from the Upper Cretaceous allowed us to examine, describe and illustrate its morphology and test its position within the family Endomychidae. The results of our phylogenetic analysis based on a morphological dataset indicate that *Cretostenotarsus striatus* new genus and species described here belongs to the subfamily Endomychinae, therefore representing the oldest known member of the endomychine complex [7] or “Higher Endomychidae” [4]. Although *Cretostenotarsus striatus* shows a second tarsomere only weakly lobed, it represents the oldest example of the pseudotrimerous tarsal configuration characteristic for the endomychine complex and family Coccinellidae (ladybugs). The abundance and diversity of members of the merophysiine complex or “basal Endomychidae” in the Upper Cretaceous ambers suggest at least a Lower Cretaceous origin of this group agreeing with the estimations of McKenna et al. [14]. But the discovery here reported of a member of “Higher Endomychidae” present in the Upper Cretaceous environments pushes back in time the possible time of origin and subsequent diversification of Endomychidae, which agrees more with Toussaint et al. [32] or Cai et al. [15] estimates on Coccinelloidea origin. Our discovery will be of great value in future testing of the hypotheses of the causes and consequences of diversification of this superfamily that require a robust time-calibrated phylogeny.

## Data Availability

The data presented in this study are available in the present article.

## Appendix A. List of Morphological Characters Used in Cladistics Analyses

1. Head with occipital file: absent (0); present (1).

2. Head with antennal grooves: short (0); long (1), absent (2).

3. Antenna: 4–5/7 (0); 8–9 (1); 10 (2); 11 (3).

4. Antennal club: 3-segmented (0); 1 or 2-segmented (1).

5. Fronto-clypeal suture: absent (0); present (1)

6. Mandible with mola: well-developed, large (0); absent or reduced (1).

7. Mandible with prostheca: only setose (0); with setae and apical, sclerotized, elongate projections (1).

8. Mandible with subapical teeth/serrations (0), with at most one subapical tooth present (1).

9. Mentum: widest basally or medially (0); widest anteriorly (1).

10. Labium with mentum: flat, smooth (0); with small, triangular, setose hill placed medially (1); with large, triangular, raised area (2).

11. Labial palp with palpomere 2: subcylindrical or transverse (0); oval, inflated (1).

12. Maxillary palpomere 2 and 3 with membranous insertions on inner apical edges: absent (0); present (1).

13. Tentorium with anterior arms: separate (0); meeting medially (1).

14. Corpotentorium absent (0); present (1).

15. Anterior margin of pronotum with stridulatory membrane: absent (0); present (1).

16. Pronotum laterally: narrowly bordered (0); with wide lateral margins (1).

17. Pronotum with basal and/or lateral sulci/modifications: absent (0); present (1).

18. Prothoracic ventral antennal grooves: absent (0); present on prosternum (1); present on hypomeron (2).

19. Mesoventrite with intercoxal process bicarinate (boat-shaped): absent (0); present (1).

20. Mesocoxal cavity: open outwardly (0); narrowly closed outwardly (1).

21. Mesotrochantin: at least partially exposed (0); concealed (1).

22. Metaventrite with postcoxal lines: absent (0); present (1).

23. Tarsal formula: 5-5-5 (or 5-5-4 in male) (0); 4-4-4 (1); 3-3-3 (2).

24. Penultimate mesotarsomere: not or slightly reduced and not enclosed within lobe of antepenultimate tarsomere (0); highly reduced and partly or entirely enclosed within ventral lobe of antepenultimate tarsomere (1).

25. Pretarsal claws: simple (0); modified (1).

26. Number of functional abdominal spiracles: 7 pairs (0); 5 pairs (1).

27. Abdominal ventrite 1: without postcoxal lines (0); with postcoxal lines (1).

28. Sternite (IX) of male genital segment with lateral edges deeply, asymmetrically curved inwardly: absent (0); present (1).

29. Tegmen: without penis guide (0); with penis guide (1); tegmen absent (2).

30. Aedeagus with median lobe: nearly straight or curved (0); coiled (1).

31. Median lobe with T-shaped capsule: absent (0); present (1).

32. Median lobe with curled 1/3 of its basal part: absent (0); present (1).

33. Ovipositor without infundibulum (0); with infundibulum-like structure/sperm duct modified (1); with infundibulum (2).

34. Ovipositor with sperm duct attached: directly to spermatheca (0); to broad connection between spermatheca and accessory gland (1).

35. Ovipositor: with deeply divided basal part of coxites (1); not divided basal part of coxites (0).

36. Mesoventral postcoxal pits: absent (0); present (1).

37. Metaventral postcoxal pits (anterior margin of metaventrite, adjacent to posterior margin of mesocoxal cavities): absent (0); present (1).

38. Antennal sockets: visible from above (0); not visible (1).

## Appendix B. Morphological Matrix Scored for 29 Taxa and 38 Characters Used for Cladistics Analyses in TNT

Hobartius_eucalypti	02301001000011000000000000000000000000
Coccinella_septempunctata	02300101100100000000111111101010200000
Chilocorus_cacti	021?0101100100000000111111101010000001
Scymnomorphus_fulvus	0021010110000000000011??01101010000000
Mycetaea_subterranea	02301000010011011000101001000000000000
Holoparamecus_sp.	02311000001010001001102001000000000001
Cholovocera_beloni	01111011021010001101112001102000000001
Merophysia_sp.	01111011021010001101102001002000000001
Leiestes_seminiger	02301001000011001010101001000000000111
Phymaphora_pulchella	02301000000011001010101001000000000111
Rhanidea_unicolor	02301000000011001010101001000000000111
Cyclotoma_testudinaria	02301001000011000000001101000001010000
Meilichius_impressicollis	02301001000011001000001101000001010010
Epipocus_tibialis	02301001000011001000001101010000000110
Epopterus_sp.	02301001000011001000001101010000000110
Endomychus_coccineus	02301001000011001000001101000000000110
Stenotarsus_nobilis	02301001000011011000001101000000011110
Tharina_antennalis	02301001000011011000002001000000001110
Aphorista_laeta	12301001000011101000001101000000000110
Eumorphus_quadriguttatus	12301001000011101000001101000000000110
Lycoperdina_bovistae	12311001000011101000001001000000000110
Pleganophorus_bispinosus	0201100000001100100110100100????000000
Trochoideus_desjardinsi	02011000000011001001101001000000000000
Xenomycetes_morrisoni	02301001000011001000001001000000000110
Danascelis_elongata	02301001000011001000001001000000000110
Eidoreus_minutus	00211001020011000000011001100110100000
Chileolobius_notatus	01310101020011000200111001100110100000
Danae_sp.	02301001000011011000001101000000001000
Cretostenotarsus striatus	?2301???0001??011000?0100?0????????110

## References

[1] Shockley F.W., Tomaszewska K.W., McHugh J.V. (2009). An annotated checklist of the handsome fungus beetles of the world (Coleoptera: Cucujoidea: Endomychidae). Zootaxa.

[2] Lawrence J.F., Newton A.F., Pakaluk J., Ślipiński S.A. (1995). Families and subfamilies of Coleoptera (with selected genera, notes, references and data on family-group names). Biology, Phylogeny and Classification of Coleoptera. Papers Celebrating the 80th Birthday of Roy A. Vol. 2, Crowson.

[3] Tomaszewska W. (2000). Morphology, phylogeny and classification of adult Endomychidae (Coleoptera: Cucujoidea). Ann. Zool..

[4] Tomaszewska W. (2005). Phylogeny and generic classification of the subfamily Lycoperdininae with re-analysis of the family Endomychidae (Coleoptera: Cucujoidea). Ann. Zool..

[5] Tomaszewska W., Leschen R.A.B., Beutel R.G., Lawrence J.F. (2010). Endomychidae Leach, 1815. Handbook of Zoology, Vol. 2, Coleoptera.

[6] Crowson R.A. (1955). The Natural Classification of the Families of Coleoptera.

[7] Robertson J.A., Ślipiński S.A., Moulton M., Shockley F.W., Giorgi A., Lord N.P., Mckenna D.D., Tomaszewska W., Forrester J., Miller K.B. (2015). Phylogeny and classification of Cucujoidea and the recognition of a new superfamily Coccinelloidea (Coleoptera: Cucujiformia). Syst. Entomol..

[8] Shockley F.W., Alekseev V.I. (2014). *Glesirhanis bercioi*, a new genus and species from Baltic amber (Coleoptera: Endomychidae: Leiestinae) with a checklist and nomenclatural notes regarding fossil Endomychidae. Zootaxa.

[9] Alekseev V.I., Tomaszewska W. (2018). New handsome fungus beetles (Coleoptera: Coccinelloidea: Anamorphidae, Endomychidae) from European amber of the Upper Eocene. Palaeontol. Electron..

[10] Reike H.P., Alekseev V.I., Gröhn C., Arlt T., Manke I. (2020). First extinct species of the genus *Holoparamecus* (Coleoptera: Merophysiidae: Holoparamecinae) from Eocene amber deposits. Stud. Rep. Taxonom. Ser..

[11] Tomaszewska W., Ślipiński A., Bai M., Zhang W., Ren D. (2018). The oldest representatives of Endomychidae (Coleoptera: Coccinelloidea) from the Cretaceous Burmese amber. Cretac. Res..

[12] Li Y.-D., Tomaszewska W., Huang D.-Y., Cai C.-Y. (2022). Rhomeocalpsua torosa gen. et sp. nov., a uknique lineage of Endomychidae from mid-Cretaceous Burmese amber (Coleoptera: Coccinelloidea). Palaeoentomology.

[13] Shi G., Grimaldi D.A., Harlow G.E., Wang J., Wang J., Yang M., Lei W., Li Q., Li X. (2012). Age constraint on Burmese amber based on U-Pb dating of zircons. Cretac. Res..

[14] McKenna D.D., Wild A.L., Kanda K., Bellamy C.L., Beutel R.G., Caterino M.S., Farnum C.W., Hawks D.C., Ivie M.A., Jameson M.L. (2015). The beetle tree of life reveals that Coleoptera survived end of Permian mass extinction to diversify during the cretaceous terrestrial revolution. Syst. Entomol..

[15] Cai C., Tihelka E., Giacomelli M., Lawrence J.F., Ślipiński A., Kundrata R., Yamamoto S., Thayer M.K., Newton A.F., Leschen R.A. (2022). Integrated phylogenomics and fossil data illuminate the evolution of beetles. R. Soc. Open Sci..

[16] Grimaldi D.A., Engel M.S., Nascimbene P.C. (2002). Fossiliferous Cretaceous amber from Myanmar (Burma): Its rediscovery, biotic diversity, and paleontological significance. Am. Mus. Novit..

[17] Ross A.J., Mellish C., York P., Crighton B., Penney D. (2010). Burmese amber. Biodiversity of Fossils in Amber from the Major World Deposits.

[18] Shi C., Cai H.H., Jiang R.X., Wang S., Engel M.S., Yuan J., Bai M., Yang D., Long C.L., Zhao Z.T. (2021). Balance scientific and ethical concerns to achieve a nuanced perspective on ‘blood amber’. Nat. Ecol. Evol..

[19] Goloboff P.A., Catalano S.A. (2016). TNT version 1.5, including a full implementation of phylogenetic morphometrics. Cladistics.

[20] Nixon K.C. (2002). WinClada.

[21] Berendt G. (1845). Die im Bernstein Befindlichen Organischen Reste der Vorwelt Gesammelt in Verbindung Mit Mehreren Bearbeitet. Erster Band. Abtheilung, I. Der Bernstein Und Die in Ihm Befindlichen Pflanzenreste der Vorwelt.

[22] Menge F. (1856). Lebenszeichen vorweltischer im Bernstein eingeschlossener Thiere.

[23] Motschulsky V.I. (1856). Voyages. Lettres de M. de Motschulsky a M. Menetries. No. 3. New York le 15 Juillet 1654. Études Entomol..

[24] Klebs R. (1910). Über Bernsteineinschlüsse im allgemeinen und die Coleopteren meiner Bernsteinssammlung. Schr. Phys. Okon. Ges. Zu Königsberg.

[25] Strohecker H.F., Wystman P. (1953). Coleoptera Fam. Endomychidae. Genera Insectorum.

[26] Spahr U. (1981). Systematischer Katalog der Bernstein- und Kopal-Käfer (Coleoptera). Stutt. Beitr. Naturkd. B..

[27] Carpenter F.M. (1992). Treatise on Invertebrate Paleontology. Part R, Arthropoda 4, Volume 4. Superclass Hexapoda.

[28] Kirejtschuk A.G., Nel A. (2009). New genera and species of Cucujiformia (Coleoptera, Polyphaga) from lowermost Eocene French amber. Denisia.

[29] Ponomarenko A.G., Rasnitsyn A.P., Quicke D.L.J. (2002). Superorder Scarabaeoidea Laicharting, 1781. Order Coleoptera Linne, 1758. The beetles. History of Insects.

[30] Poinar G.O., Poinar R. (2008). What Bugged the Dinosaurs? Insects, Disease, and Death in the Cretaceous.

[31] Tomaszewska K.W. (2000). A review and a phylogenetic analysis of the genera of Leiestinae (Coleoptera, Endomychidae). Dtsch. Entomol. Z..

[32] Toussaint E.F.A., Seidel M., Arriaga-Varela E., Hájek J., Král D., Sekerka L., Short A.E.Z., Fikáček M. (2017). The peril of dating beetles. Syst. Entomol..

